# Data Integrity Issues With Web-Based Studies: An Institutional Example of a Widespread Challenge

**DOI:** 10.2196/58432

**Published:** 2024-09-16

**Authors:** Blandine French, Camilla Babbage, Katherine Bird, Lauren Marsh, Mirabel Pelton, Shireen Patel, Sarah Cassidy, Stefan Rennick-Egglestone

**Affiliations:** 1 School of Psychology University of Nottingham Nottingham United Kingdom

**Keywords:** web-based research, web-based studies, qualitative studies, surveys, mental health, data integrity, misrepresentation

## Abstract

This paper reports on the growing issues experienced when conducting web-based–based research. Nongenuine participants, repeat responders, and misrepresentation are common issues in health research posing significant challenges to data integrity. A summary of existing data on the topic and the different impacts on studies is presented. Seven case studies experienced by different teams within our institutions are then reported, primarily focused on mental health research. Finally, strategies to combat these challenges are presented, including protocol development, transparent recruitment practices, and continuous data monitoring. These strategies and challenges impact the entire research cycle and need to be considered prior to, during, and post data collection. With a lack of current clear guidelines on this topic, this report attempts to highlight considerations to be taken to minimize the impact of such challenges on researchers, studies, and wider research. Researchers conducting web-based research must put mitigating strategies in place, and reporting on mitigation efforts should be mandatory in grant applications and publications to uphold the credibility of web-based research.

## Introduction

Web-based research, such as web-based surveys or qualitative interviews via videoconferencing platforms, has grown in popularity and usability in the last 2 decades but more specifically since 2020 and the COVID-19 global pandemic [1-3]. Web-based studies have enabled researchers to continue conducting research studies when in-person testing was not possible [4], facilitating the recruitment of large samples, specific samples, or populations often excluded from research (described as underserved communities, or “hard to reach”) [5,6]. It also offers other benefits such as minimizing recruitment costs [7] or allowing for anonymity, often favored by specific populations [8]. Web-based anonymity is attractive for certain groups as the researcher does not meet the participants and may even allow participation from individuals who might otherwise feel excluded from research; however, this lack of in-person validation poses a distinct threat to the validity, quality, and integrity of the data [9].

While web-based research has been heavily used by researchers due to its many advantages, this increased use has also led to researchers questioning whether their web-based participant pool is genuine. Web-based research describes “any research involving the remote acquisition of data from or about human participants using the internet and its associated technologies” [10], including both quantitative (eg, surveys or questionnaires) and qualitative methodologies (eg, focus groups and interviews). The following 5 categories of behavior, by participants and others, can significantly impact data quality and integrity in web-based studies:

1. Nongenuine participants: participants lying about their lived experiences or identity.
2. Repeat responders: participants taking part more than once.
3. Misrepresentation: exaggeration of specific details.
4. Lack of engagement: participants answering quickly, not paying attention and not reading the questions fully.
5. Bots: automated software application that performs repetitive tasks over a network.

These categories encompass not only different methodologies (qualitative and quantitative) but also different samples (general population and specific samples). While historically web-based studies were used by behavioral scientists (often aimed at the general population), health research (often aimed at specific groups) has also increasingly transitioned online [11]. Primarily issues relate to repeat responders, lack of engagement, and bots [12,13]. To remedy and minimize these issues many “secure” web-based behavioral platforms have been developed, such as Amazon Mechanical Turk [14] or Prolific Academic [15]; however, their reliability is often contested [12,16]. These issues have a significant impact on data integrity with lack of engagement, for instance, as high as 40% [17].

When conducting our research with specific participant groups with a range of health and neurodevelopment conditions, we have often experienced issues with nongenuine participants, repeat responders, and misrepresentation. Many studies indicate that a subset of participants in web-based surveys tends to falsely assert eligibility to obtain entry into the study, particularly when the incentive for participation is monetarized [18,19]. Hydock (2017) [20] reported that a “small but nontrivial portion of participants in online survey studies misrepresented their identity for the chance of financial gain.”

This publication focuses solely on health web-based research and its most common barriers, namely, nongenuine participants, repeat responders, and misrepresentation. Challenges and strategies to overcome these difficulties are presented, most relevant to researchers in this field of work, which requires necessary caution given the complex health conditions and participants being regarded.

The motivations for repeat responders or nongenuine participants are varied, including monetary incentive [19,20], accessing a special intervention or treatment [21], or be politically motivated [22]. Indeed, 1 study experienced purposeful interference and registration in their study from specific groups with vested interests [22]. Finally, repeat registration can also be due solely to human error, for example, not realizing that they had already signed up, though insincere behaviors could be to receive further financial incentives.

Many studies have reported the significant impacts of these issues on their studies. In a series of 3 studies, Chandler and Paolacci [19] found that between 3% and 40% of participants misrepresented themselves, for example, saying that they were US residents when they were not. Bowen et al [23] recorded the numbers of repeated responses (627/1900, 33%) and demonstrated the wide extent by classifying them into 4 categories: infrequent (2-5 responses from same person), persistent (6-10), repeater (11-30), and hackers (45-67). These issues have also been reported in qualitative web-based interviews [24-26], for example, Roehl and Harland [24] reported 5 out of 14 participants as inauthentic. Finally, many studies report great difficulties after advertising on social media. Salinas [27] found that only 9.8% (50/512) of respondents from Facebook adverts were genuine while Pozzar et al [28] reported that after 7 hours of advertising their study on Twitter, 94.5% (256/271) were inauthentic and 16.2% (44/271) of them showed evidence of bot automation.

The impact of these issues on data integrity is significant. Chandler et al [29] demonstrated that inauthentic data have a significant impact on experiments—by increasing between-group variance, on individual differences—by providing false information, and on the association between variables—by suppressing, inflating, or reversing correlation. For example, Ysidron et al [16] found that nongenuine participants in a study on diabetes (150/307, 49% of the sample) reported significantly poorer physical and mental health issues than the clinical group, suggesting substantial exaggeration of adverse health. This leads to incorrect conclusions, potentially creating inappropriate recommendations for both practice and future research. The impact of these issues also greatly increases research costs both in terms of financial cost and time [30].

After having experienced many of these issues in their health research studies, the research team collated their individual experiences. Strategies and tips were exchanged, leading to institution-wide presentation and awareness to inform any future studies and initiating institutionally recognized strategies. This publication reports a sample of case studies experienced within our UK-based institution at the University of Nottingham and reflections on the methodological challenges and strategies extracted from the high-quality publications in the area.

## Approach

### Case Studies

We present 7 case studies experienced within our institution, all relating to mental health research. It is not possible to summarize all issues experienced within our institution in 1 paper. Consequently, this list is not exhaustive of issues experienced but rather it describes some examples of the breadth of this issue and how it impacted each research team. Table 1 shows the different case studies included in this manuscript.

**Table 1:** University of Nottingham case studies.

Case study	Methodology used	Category of issues experienced	Participants impacted, n (%)	Impact on study
1	Qualitative: interviews	Nongenuine Participants Repeat responders	8/12 (66.67) were nongenuine data.	Loss of funds, inability to finish project or publish, time wasted by PI^a^ and researcher to identify data.
2	Randomized controlled trial	Repeat responders	100/1123 (8.9) accounts were suspended due to repeat registration.	Human resources to develop and administer repeat registration protocol and suspend accounts suspected of repeat registration. Additional burden and distress for participants who repeatedly registered in error.
3	Randomized controlled trial	Nongenuine participants Repeat responders	Approximately 482/483 (99.79) were suspected as nongenuine participants.	Impact on study researchers in terms of increased workload but it did not impact on recruitment figures.
4	Quantitative: survey	Nongenuine participants Repeat responders	349/391 (89.26) were suspected as nongenuine participants.	The process of cleaning the data was difficult in decision-making, time-consuming, and led to delays in completing the project. Reluctance to widely advertise afterward.
5	Qualitative: interviews	Nongenuine participants	54/54 (100) were suspected as nongenuine; 7 were identified as nongenuine. No genuine participants were recruited.	Loss of time, had to widen recruitment to the general population to deliver the project.
6	Qualitative: focus groups	Nongenuine participants; repeat responders	Approximately 115 suspected nongenuine participants applied. 83% of selected participants were nongenuine.	Loss of funds, loss of time, and loss of data integrity.
7	Quantitative: administration of online task	Nongenuine participants	20/31 (64.52) nonautistic participants consented to the study were nongenuine.	Loss of comparison group, unable to fully deliver funder expectations, time, and stress.

^a^PI: principal investigator.

### Case Study 1: Web-Based Interviews on Cognitive Behavior Therapy and Attention-Deficit/Hyperactive Disorder

#### What Happened?

The study aimed to establish the experience of cognitive behavior therapy (CBT) in adults with attention-deficit/hyperactive disorder (ADHD) through semi-structured interviews as part of a masters’ student project. The research study invited adults with ADHD from the principal investigator’s research database and received multiple and prompt expressions of interest. She interviewed 12 participants in a couple of weeks but after finishing the data collection, she felt that there was “something wrong” with some interviews.

#### How Did You Find Out?

Out of 12 interviews, 8 were suspicious. The interviewer felt that the responses were very brief and not in-depth, some stories were very similar if not identical, and the participants followed a similar pattern. All 8 interviews had similar format of Gmail email addresses, refused to put their camera on, related similar stories, had identical non-British accents, and were actively asking about payments. In addition, from our decade-long experience in this topic, we know that most adults with ADHD have very difficult experiences with CBT, and this group all reported very positive experiences of CBT which is very unusual. As only participants from the database had been invited, all participants (n=6) who were not on the database were excluded automatically after asking how they heard about the study. The remaining 2 narratives were too similar to some of the excluded ones and were therefore also excluded.

#### How Did It Impact Your Research (Financial and Time Costs)?

The whole student project was compromised by this issue. As participants were paid £20 (US $26) per interview, no extra budget was available to conduct additional interviews as well as no extra time within the constraint of the master’s dissertation submission. The analysis had to be conducted on 4 interviews alone, which was acceptable for the dissertation purposes (after explaining the issue to the head of teaching) but not for publication. The student was very disappointed by the amount of time that had been wasted on these and the impact it had on her opportunity to get her project published.

### Case Study 2: Randomized Controlled Trials, Narrative Experiences Online

#### What Happened?

The Narrative Experiences Online study conducted 2 definitive pragmatic web-based trials of a web-based digital health intervention, which provided access to a collection of mental health recovery narratives. To participate in the trial, UK residents completed a web-based informed consent form and registered a web-based account using a personal email address. Formal identity verification was not required by our regulator, and we chose not to use it, to avoid contributing to paranoia for a trial population who had a personal experience of psychosis (for the Narrative Experiences Online Trial) [31]. We were prepared for the possibility of repeat registration of accounts due to published accounts of previous web-based trials [21], and so our protocol included an outline procedure to suspend accounts suspected of repeat registrations [31]. As recruitment to our trials progressed, we observed a range of instances of suspected repeat registrations. We decided to formalize our decision-making procedures on account suspension.

#### How Did You Find Out?

The web application that delivered our trials included an administrative control panel providing access to information about all registered accounts. Through regular monitoring, we saw instances of sequential registrations, using email addresses that appeared related, with little time between each registration, or from the same IP address. We communicated with some participants and learnt that some instances were accidental repeat registrations due to confusion with trial procedures and others were deliberate (including to obtain payment vouchers). Many participants did not respond to our messages. In some cases, there were legitimate reasons for these indicators. These included registration by more than 1 person from the same household, often on the same device, where trial information had been shared among the household.

#### How Did It Impact Your Research?

We spent a substantial effort on a protocol for decision-making about account suspensions (Multimedia Appendix 1), which was necessary given ambiguous decision-making (eg, where some participants did not respond to contact). Our protocol developed by the research team was amended and authorized by a trial management group and subsequently authorized by a program steering committee. We produced our protocol for the important purposes of (1) supporting trial integrity, and (2) enabling ethical conduct in communication with participants. Enacting the protocol required a substantial amount of time on behalf of administrators, who collected information on possible repeat registrations in “Repeat Registration Reports” (Multimedia Appendix 2). It also required time from the decision makers who reviewed those reports. For analytical integrity, further effort was spent on articulating a modified intention-to-treat principle, which excluded accounts suspended due to repeat registrations [32], and on developing a modified CONSORT diagram to report on suspensions [32,33].

### Case Study 3: Randomized Controlled Trial, Mindful Life-Well at Work

#### What Happened?

The Mindful Life-Well at Work study is a randomized controlled trial (RCT) assessing whether Mindfulness-Based Cognitive Therapy—for Life is more effective than stress reduction psychoeducation. Participants are recruited from health care, social care, and teaching sectors. To participate in the trial, clinical staff who receive the study flyer circulated by their participating NHS trust sites complete a web-based eligibility screening and informed consent form. All forms and questionnaires are completed independently by participants using the web-based REDCap (Research Electronic Data Capture) platform. During the second recruitment wave we received an influx of emails; 483 emails were received over 5 days. The emails were sent out at strange times (eg, 3 AM) and a large number of emails were received within a few minutes of each other. The number received was considerably larger than in earlier recruitment periods.

#### How Did You Find Out?

Initially, the study researchers did not suspect any dubious activity. However, on the second day when a study researcher was recording the data for the noneligible participants, the researcher noticed that all inclusion or exclusion criteria had been selected for most cases. This was peculiar, as data from previous participants implied that usually 1 or 2 criteria were selected. Upon closer scrutiny, they noticed that the email account names did not match the names provided. The study team also observed that signatures on the consent forms indicated that the same person was completing them. The first batch of emails was sent out from Gmail and the second batch was from Hotmail. The data on REDCap suggested that many tabs had been opened at the same time and forms completed one at a time, that is, submitted each consent form approximately 5 seconds after each other.

#### How Did It Impact Your Research (Financial and Time Costs)?

Despite the study team feeling quite convinced that these were not emails from actual participants, they investigated further to ensure that actual participants were not being missed. The senior database manager was consulted for advice. He informed the study team that as IP addresses were not collected because they are identifiable; all cases would need to be treated as actual participants until it was verified that they were not genuine. All those not deemed to be eligible were emailed and provided with alternative sources of support. All those who had consented were emailed to ask whether they would be happy to be contacted when program dates became available. Of the 483 emails sent out only 1 reply was received. In terms of resource implications, this required additional time from study researchers as they had to go through numerous emails, ascertain whether it may be a fake participant, record this on a separate log, and reply to all emails. We were pleased that this had been identified early on before any nongenuine participants were randomized to the trial.

### Case Study 4: Web-Based Survey to Assess the Acceptability of SPARX

#### What Happened?

This project assessed whether any changes needed to be made to SPARX [34], a game developed in New Zealand that delivers CBT to young people with low mood and anxiety, ahead of a pilot and feasibility RCT in the United Kingdom [35]. To do this, a Joint Information Systems Committee web-based survey was promoted via relevant networks and organizations, including social media. Our target was to reach 100-200, 11- to 19-year-olds. The survey was designed so that consent (for those older than 16 years) or consent (from a parent or legal carer) and assent (from those younger than 16 years) were initially obtained, which would direct young people to complete the study survey. After consent, on the final page of the survey, young people were directed to another web-based survey via a link where they could register for a prize draw. Questions were open-ended, asking the age of the responders, what they liked about playing computer games (question 8), and what they thought the advantages of e-therapy were (question 14). In total 415 young people gave consent; 391 participants completed the web-based survey and 372 registered for the prize draw.

#### How Did You Find Out?

The first suspicious activity noted was the speed of recruitment, receiving 299 responses to the survey in 1 day. On collating responses, it became clear that many participants (184/391, 47%) were not eligible (ie, being outside of the age of 11-19 years). Responses to question 8 were exact, case-sensitive duplicates of other responders, with 63.8% (132/391) answers being replicas and not answering the question. From this, only 28.5% (59/207) eligible responses were not noted as suspicious. A further 17 responses were noted as suspicious as when reviewing question 14. Many responses seen in question 8 were also made here and did not make sense in the context of the question asked. For example, the words “the Forest” appeared throughout the survey responses. From all responses to the survey, we were left with 10.7% (42/391) responses believed to be genuine.

#### How Did It Impact Your Research?

The process of cleaning the survey data was time-consuming and led to delays in completing the project. In addition, despite wanting to recruit more young people due to the team being unsure where the suspicious activity was coming from, the team was reluctant to continue pushing for recruitment. Furthermore, it has been difficult to decide whether these data should be published as there are no guarantees that the final included responses are genuine. Perhaps most importantly, given the reliance on this research in informing an upcoming RCT, caution was required in how the data could be applied to the study design. Finally, in reflecting on the issue, the experience brought about interesting feelings about one’s trust in the data that they have collected.

### Case Study 5: Web-Based Interviews on Self-Harm in Transgender People

#### What Happened?

We recruited transgender people for a web-based interview study regarding factors for self-harm. The study offered a £10 (US $13) voucher for transgender views on an existing research tool and was advertised on social media (ie, Reddit), alongside other recruitment pathways. Once the study was advertised online, we received more than 50 responses expressing interest in 24 hours. The sheer number of respondents raised suspicions as respondent numbers far exceeded previous recruitment drives. At this stage, it was noted that all emails, without exception, were gmail.com accounts and followed the same pattern: first name, last name, and then a series of numbers. Also, some participants used terminology which, in our experience, is not used by the transgender community (ie, “I am a transgender”). These raised suspicions, but we did not want initial suspicions to result in genuine participants being missed, so follow-up emails were sent with study and consent details to all.

#### How Did You Find Out?

The nature of the study required potential participants to complete a well-being plan alongside standard informed consent. This entailed providing contact details of their general practitioner (GP: family doctor) and a trusted person and the address from which they will take part. People who regularly take part in self-harm research are familiar with ethical requirements and complete necessary forms in an appropriate manner. However, here respondents either did not complete the well-being plan or omitted necessary information. Some did complete well-being plans, but these raised further suspicions that these were fake participants. For example, GP surgery details included a mobile phone and a Gmail address, neither of which is typical for UK GP surgeries. Because of this, we performed cursory Google searches of both the GP and home addresses provided in the well-being plan to ensure that they were legitimate addresses. In all cases, it transpired that the addresses provided were either entirely false (ie, did not exist at all) or were commercial addresses. In one instance, for example, the “home address” was a hotel in central London and another was an industrial property. These addresses may be temporary residential properties or used during work hours to take part; however, they did not feel authentic. The false addresses, false or suspicious GP details, poorly completed well-being plans, suspicious terminology, and all respondents using Gmail accounts with similar email addresses left the researcher believing that these respondents were inauthentic, and they were not recruited to the study.

#### How Did It Impact Your Research (Financial and Time Costs)?

The process was disheartening, and the researcher spent hours responding to and identifying nongenuine responses, but early suspicions meant that the researcher avoided wasting significant time and resources interviewing and having discarded data. However, no genuine participants were recruited for this study, and we had to widen the study to the general population.

### Case Study 6: Web-Based Focus Group on Inclusion at Work for Autistic Adults

#### What Happened?

We advertised for autistic participants to take part in a web-based focus group. The advert was posted by a project partner on Twitter. Approximately 150 potential participants responded within 24 hours, which was more than anticipated and more than we could invite to take part. The advert was removed, and the research team was suspicious that there were some nongenuine respondents. To tackle this, we asked respondents for demographic and diagnostic information and their motivation for being involved. We received 114 responses, the majority of which we believe were inauthentic. We invited 6 respondents to the focus group who we thought were most likely genuine participants.

We ran the web-based focus group with 6 participants for 1 hour. All participants turned on their cameras and provided verbal answers to the discussion questions. However, responses from 5 out of the 6 participants were general and provided little depth or personal perspective. When prompted, they did not expand their points or give specific examples. All 3 researchers present strongly suspected that 5 out of 6 participants did not have lived experience of autism. Although this was impossible to prove, the research team has more than 30 years of combined experience working closely with autistic adults and this experience led them to suspect that these participants were being insincere about their lived experience.

#### How Did You Find Out?

The large volume of volunteers in the short time window was the first indication that the initial recruitment phase had attracted nongenuine participants. This was confirmed by the request for further information as many motivation statements were duplicated between respondents, or were superficial, textbook-like responses. Like previous case studies, most respondents had Gmail addresses in the form of first name, last name, and a random number. Despite carefully selecting participants based on the quality of their responses and removing responses where inconsistent or suspicious information was provided, we were still not confident that our included participants were genuine.

#### How Did It Impact Your Research (Financial and Time Costs)?

Participants received a £30 (US $39) voucher, regardless of their contribution. We experienced an ethical dilemma about whether to include the data. Given we had no proof of inauthentic activity, we chose to analyze all the data and also collect and include additional data to identify converging themes from a wider range of people and experiences. Aside from potentially compromising our data, this experience was financially costly (paying additional participants), time-consuming (dealing with many emails, filtering and identifying genuine responses, and running and analyzing data from inauthentic focus groups), and had an emotional impact on the researchers.

### Case Study 7: Exploring Patterns of Self-Harm in Autistic and Nonautistic Adults

#### What Happened?

We recruited autistic and nonautistic adults for a web-based interview study comparing proximal and distal risk markers for self-harm between the 2 groups, using the Card Sort Task for Self-Harm. Participants were offered a £10 (US $13) voucher for participating. We advertised the study through social media, charities, and volunteer research databases. Although many genuine participants signed up for our study, we became suspicious that a small group of participants recruited from 1 mental health research platform were not genuine participants.

#### How Did You Find Out?

Our study required participants to have an initial meeting with the researcher to complete a web-based demographics questionnaire to check eligibility, create a well-being plan, and obtain informed consent to take part. The well-being plan (identical to the one in case study 6) entailed providing multiple contact details and addresses. Several indicators raised our suspicions that some participants were not genuine. First, several indicators raised our suspicions that some participants were not interested in the research process with initial emails focusing on payment. Participants appeared to lack knowledge about the subject and could not provide details of their experiences after being probed. Second, there was evidence of participants attempting to register and take part in the study more than once such as refusing to turn their camera on. Despite this, it was clear to the researchers that the person had already taken part in the study. Furthermore, it was also clear that demographic data had been entered more than once, provided neighboring or fake addresses, fake phone numbers, and incomplete information (eg, no details of health care provider or trusted person to contact). Together, these indicated a small group of participants who were attempting to register and take part in the study more than once, presumably for financial gain.

#### How Did It Impact Your Research (Financial and Time Costs)?

The small group of participants who attempted to take part in the study more than once, and provided unreliable data, had to be excluded from the data set. It took a significant amount of time to be confident that our study had been infiltrated by nongenuine participants. By the time we had identified an issue and taken action, we had run out of time and funding to complete data collection for the nonautistic group. This meant that we had to revise the aims of the research to explore patterns of self-harm in the autistic group only—we were not able to make comparisons with nonautistic adults as we had planned. We had to submit a report explaining this to the funder on completion of the study.

The multiple case studies reflect a snippet of some of the institutional experiences within our health research teams. While we could not incorporate all the different case studies presented to us within our institution as they are far too many, these brief experiences summarize the many different ways that it can impact research project from multiple different methodological approaches.

## Methodological or Research Challenges and Strategies

### Overview

To minimize the impact of issues with web-based studies (qualitative or quantitative), it is essential to have a systematic process for “determining the level of suspicion required to remove potentially unreliable data” [36]. This includes many strategies that have been summarized in publications [36-38] such as a framework outlining the importance to Reflect, Expect, Analyze, and Label [36].

To think about the different challenges, it is important to think about these issues in relation to the whole research cycle and ask different questions at different stages of the research process [25]:

1. During funding application: What problems are likely to occur? What resources do I need to include to manage these problems?
2. Before recruitment: What is likely to happen? Can I develop a protocol that identifies and considers as many potential issues as possible?
3. During recruitment: Can I verify that the participant met the inclusion criteria? How confident am I in this information?
4. During data collection: Was the participant hesitant or flustered when asked probing questions for additional detail? What were my first impressions of honesty in my reflexive journal? Did I note any nonverbal cues that might be a clue to participant dishonesty?
5. During data analysis: Did I find places where the participants contradicted themselves? Were a participant’s answers detailed enough that the participant seemed knowledgeable about the topic?

There are many noteworthy strategies put forward in this literature [36-38] and we bring together these challenges and strategies with our case studies to support applications for how one might implement them within their web-based research.

### Pre–Data Collection

Pre–data collection refers to 2 different stages of the research cycle: ethics application and recruitment.

#### Challenges

The challenges experienced at the ethics application stage involve thinking in advance about what the potential threats to data integrity are. This will apply to threats throughout the research project and how the research team plans on dealing with these. In terms of recruitment, these threats could be multiple sign-ups from the same participants or nongenuine participants signing up (pretending to have specific health conditions or eligibility criteria). Some of the patterns that have been observed in detecting those include numerous entries very quickly, numerous consecutive entries with the same email format (name.surname999@gmail.com), entries from countries outside the recruitment area, similar IP addresses, refusal to provide a phone number or other key details, fast response to communication, or inconsistencies.

#### Strategies

We present some of the strategies our research team has put in place. It is important to note that some strategies might not be accepted by certain ethics committees and lived-experience groups and liaising with them is essential prior to recruitment.

Some useful strategies that we have put in place and found useful include the following:

1. *Protocol*: Creating a protocol before recruitment mitigating these issues as much as possible and where possible, coproduced with a lived-experience group to ensure the acceptability of the chosen strategies. To develop this protocol, it is useful to think about the following questions: How will you deal with inauthentic data? And how will I establish inauthentic data?
2. *Transparency*: Include all steps to be taken for nongenuine or multiple registration in the participant information sheet.
3. *Social media*: Close or strongly limit recruitment from particular social media platforms. Once your advert is on public platforms, you can no longer control how far and where it is distributed with many people sharing it, leading to nongenuine registrations. If advertising on social media, do not use terms such as “payment” or “gift cards” in adverts.
4. *Incentives*: One strategy is to not offer any incentives which will not be attractive to any nongenuine participants. Alternatively, offering “in-kind” incentives that would only be attractive to your target group can be helpful. For example, in a sleep study, participants were offered a sleep training course upon completion.
5. *Technical considerations*: Monitor responses from the same IP addresses. Installing cookies detector and CAPTCHA. Creating single-use links for each response. Having experts, for example, computer science, social media, and database experts as part of the team.

### During Data Collection

#### Challenges

It is also important to check these issues once participants are recruited onto the study. Our team has experienced many challenges at this stage despite inputting strategies before data collection. Some of the challenges included people providing contradicting or inconsistent responses (between eligibility criteria and consent form), providing similar stories or responses to the study multiple times, vague answers or those who cannot elaborate when prompted, and shorter than average time in responses; in qualitative studies, refusing to put cameras on video calls, poor quality, or technical problems; and in quantitative studies, giving straight-line answers, high levels of nonresponse, answers that do not make sense, and empty free text boxes.

#### Strategies

Following recruitment but before data collection, a few strategies can be put in place.

1. *Interviews*: Asking participants to do a brief phone interview to check eligibility criteria before taking part. Providing a valid phone number as well as asking to briefly turn on the camera will often rebut nongenuine participants.
2. *Identity*: For participants with specific conditions, insider knowledge is useful—asking them to describe their lived experiences could help identify nongenuine participants. In addition, asking for a valid ID or documentation on the condition (for those who have received a diagnosis) can help minimize multiple and nongenuine responses.

It is also important to have strategies in place as data are collected.

1. *Data*: Keep checking data as they come through. Check for survey or interview duration, duplicate responses, or look for inconsistencies in responses. Keep an eye out for responses that do not “feel right.” Roll out recruitment gradually to have time to check and stop any potential issues with the data collection process.
2. *Implement data screening*: Follow the data-screening plan and the protocol on reimbursement and data inclusion.
3. *Reflexive notes*: Keep track of your decision-making process and any challenges occurred at this stage for full transparency.

### Post–Data Collection

#### Challenges

Once data collection is undertaken and multiple checks have been performed, it is also important to think about what will become of any data that you establish as nongenuine. For example, it is possible that participants sign up multiple times or do not complete certain questions, but these might be due to legitimate reasons. Issues around transparency in reporting the extent of data removed can arise in terms of how much to disclose in limited word count or potential concerns from reviewers.

#### Strategies

1. *Checking*: Contacting participants who responded multiple times or whose responses seem inconsistent to give them a chance to explain any personal difficulties with the study. Conducting regular debriefs with the research team to make decisions as a group and support the researchers’ well-being.
2. *Incentives*: Aside from offering no incentives or in-kind incentives, check data before giving incentives, avoiding automated payments.
3. *Transparency*: Notify ethics or funder of issues with nongenuine participants and their impact on the study. Be transparent in reports or papers, establishing the ratio of this impact is important and should be disclosed in further publications.

It is important to note that while these strategies are often very useful, they also present challenges to researchers in terms of inclusivity, accessibility, or engagement. There are no foolproof strategies and researchers need to assess the benefits and limitations each provides.

## Discussion

This publication reflects the significant impact and presence of issues with web-based studies by disclosing some of our experiences and the challenges faced with the strategies implemented. Primarily focused on mental health research, our experiences report a range of difficulties with repeated responders and nongenuine participants. Careful considerations and strategies are presented to help mitigate the threats these experiences can have on data integrity. These experiences and suggestions are given alongside those put forward in the literature, along the research cycle to give clear suggestions for consideration in future web-based research. We have shown that the issues around web-based data collection are broad and widely experienced, and our case studies reflect the increasing threat to data integrity. While we were not able to capture all the case studies in this publication, we know of at least 20 individual cases experienced on top of our reported studies.

It is important to note that a lot of the strategies presented have limitations. For example, tracking IP addresses can be easily changed by the use of a VPN which prevents the researcher from tracking genuine country of origin and it is also impossible to track through Gmail accounts [39]. In addition, it restricts members of the same households from joining, potentially excluding genuine participants. Other strategies can also be perceived as unethical or counterproductive. For instance, not recompensing participants for their time and effort [40] or assessing eligibility either in person or on the phone [41] might not be acceptable for certain participant groups and does not capitalize on the full benefits and appeal of web-based research. Some strategies are also not friendly to all groups. For example, while some conditions can be easily “confirmed” with a diagnostic letter, many mental health conditions can be trickier to demonstrate or are stigmatized conditions that may prevent people from getting involved (eg, anxiety and self-harm). Questioning participants’ lived experiences through some form of justification might be extremely insensitive and unacceptable.

Many considerations must be taken when thinking about strategies through the research cycle. Some strategies can become quickly obsolete, for example, as technology evolves, new ways of bypassing existing strategies are always emerging. For example, the recently created platform of ChatGPT can easily replicate a believable experience of living with certain conditions. In addition, experienced users have been known to exchange tips on social media platforms on how to bypass study criteria and maximize rewards over engagement (eg, ProlificAC on Reddit).

In terms of the research cycle, it is important to remember that it impacts all areas of the research cycle (from grant application to writing up) and to think about all the different threats to data integrity as early as possible. Most strategies also require resources (time and money). Hence, appropriate resources must be requested in grants, or provided directly by institutions from central funding. Institutions might consider putting in place structural support for researchers doing web-based studies, such as through continuous professional development of staff that support studies, for example, research librarians, and research data managers.

In addition, we are unaware of any study yet that reports successfully addressing all issues of nongenuine participants, despite the efforts of developing strategies and seeking ethical approval. In a survey of fraud detection, Ballard et al [8] implemented 9 different strategies to detect and deter nongenuine responses and their final results demonstrated that only 38.9% (161/414) of responses were genuine.

These issues impact the quality and integrity of the data and add significant financial and time costs to a research project. In terms of financial cost, projects may waste funding on paying nongenuine participants. Timewise, hours and days are spent putting strategies into place and checking the data, which takes away essential time spent on other aspects of the project. It also has a cost in terms of mental well-being. We have found, as researchers, that spending time investigating sensitive health care topics to find out that participants have misrepresented their experiences, invalidating others’ experiences, makes you doubtful of the data and is potentially heartbreaking. An agreed decision-making protocol can facilitate the process of including or excluding participants and reduce pressure on researchers to make decisions, which could impact study findings [38,42]. It is also important to note that issues affecting data integrity have a significant impact on participants as well as researchers. In our experience of conducting advisory groups and focus groups to identify key research topics, having even just 1 member in this group pretending to have a condition and listening to other’s experiences can be very distressing, and navigating the facilitation of these situations requires high-level expertise. Especially if the extent of the fraud leads to nonpublication of results and essentially what could be perceived as a “waste of time.”

Finally, as suggested by recent and insightful publications on the impact of these issues on specific groups, it is tricky but essential to strike a balance between data integrity and participants’ vulnerability [38]. It is essential to maintain trust with participants, especially from potentially vulnerable backgrounds in the case of health research. Therefore, exclusion should be dealt with very carefully as while it is important to ensure that nongenuine participants’ results are not included in the analysis to minimize its impact on results, it is also essential to give genuine participants the benefit of the doubt and not exclude genuine mistakes or difficulties in communications.

In conclusion, many important considerations need to be given throughout the project to minimize, as much as possible, the impact of multiple responses, bots, and nongenuine participants on our data. This is not easy and while many strategies exist and are useful, their efficacy highly depends on the project’s methodology and population of choice. Careful considerations need to be taken when implementing these strategies, ensuring that they are acceptable and feasible within the remits of each project. This is not a quick process and involves time and resources. However, these are essential in conducting web-based studies as without these checks, it is very unlikely that the data collected will be reliable and representative. Reporting how these risks have been mitigated should become compulsory in upcoming grants and publications to ensure data integrity and credibility for web-based research.

## Appendix

Multimedia Appendix 1 Repeat registration procedures.

Multimedia Appendix 2 Repeat registration report.

## Abbreviations

ADHD: attention-deficit/hyperactive disorder
CBT: cognitive behavior therapy
GP: general practitioner
RCT: randomized controlled trial
REDCap: Research Electronic Data Capture

## References

[1] Hlatshwako TG, Shah SJ, Kosana P, Adebayo E, Hendriks J, Larsson EC, Hensel DJ, Erausquin JT, Marks M, Michielsen K, Saltis H, Francis JM, Wouters E, Tucker JD (2021). Online health survey research during COVID-19. Lancet Digit Health.

[2] De Man J, Campbell L, Tabana H, Wouters E (2021). The pandemic of online research in times of COVID-19. BMJ Open.

[3] Jones A, Caes L, Rugg T, Noel M, Bateman S, Jordan A (2021). Challenging issues of integrity and identity of participants in non-synchronous online qualitative methods. Methods Psychol.

[4] Kramer J, Rubin A, Coster W, Helmuth E, Hermos J, Rosenbloom D, Moed R, Dooley M, Kao Y, Liljenquist K, Brief D, Enggasser J, Keane T, Roy M, Lachowicz M (2014). Strategies to address participant misrepresentation for eligibility in Web-based research. Int J Methods Psychiatr Res.

[5] Routen A, Bambra C, Willis A, Khunti K (2022). Hard to reach? Language matters when describing populations underserved by health and social care research. Public Health.

[6] Eysenbach G, Wyatt J (2002). Using the Internet for surveys and health research. J Med Internet Res.

[7] Lefever S, Dal M, Matthíasdóttir Á (2007). Online data collection in academic research: advantages and limitations. Br J Educ Technol.

[8] Ballard AM, Cardwell T, Young AM (2019). Fraud detection protocol for web-based research among men who have sex with men: development and descriptive evaluation. JMIR Public Health Surveill.

[9] Gagné N, Franzen L (2003). How to run behavioural experiments online: best practice suggestions for cognitive psychology and neuroscience. Ubiquity Press.

[10] The British Psychological Society Ethics guidelines for internet-mediated research.

[11] Mortensen K, Hughes TL (2018). Comparing Amazon's mechanical turk platform to conventional data collection methods in the health and medical research literature. J Gen Intern Med.

[12] Peer E, Rothschild D, Gordon A, Evernden Z, Damer E (2022). Data quality of platforms and panels for online behavioral research. Behav Res Methods.

[13] Yarrish C, Groshon L, Mitchell J, Appelbaum A, Klock S, Winternitz T, Friedman-Wheeler D (2019). Finding the signal in the noise: minimizing responses from bots and inattentive humans in online research. Behavior Therapist.

[14] Buhrmester M, Kwang T, Gosling SD (2011). Amazon's mechanical Turk: a new source of inexpensive, yet high-quality, data?. Perspect Psychol Sci.

[15] ScienceDirect Prolific.ac—a subject pool for online experiments.

[16] Ysidron DW, France CR, Yang Y, Mischkowski D (2022). Research participants recruited using online labor markets may feign medical conditions and overreport symptoms: caveat emptor. J Psychosom Res.

[17] Osborne JW, Blanchard MR (2010). Random responding from participants is a threat to the validity of social science research results. Front Psychol.

[18] Dennis SA, Goodson BM, Pearson CA (2020). Online worker fraud and evolving threats to the integrity of MTurk data: a discussion of virtual private servers and the limitations of IP-based screening procedures. Behav Res Account.

[19] Chandler JJ, Paolacci G (2017). Lie for a dime: when most prescreening responses are honest but most study participants are impostors.

[20] Hydock C (2018). Assessing and overcoming participant dishonesty in online data collection. Behav Res Methods.

[21] Murray E, Khadjesari Z, White IR, Kalaitzaki E, Godfrey C, McCambridge J, Thompson SG, Wallace P (2009). Methodological challenges in online trials. J Med Internet Res.

[22] Briggs L, Fronek P (2018). Faking participant identity: vested interests and purposeful interference.

[23] Bowen AM, Daniel CM, Williams ML, Baird GL (2008). Identifying multiple submissions in Internet research: preserving data integrity. AIDS Behav.

[24] ProQuest Imposter participants: overcoming methodological challenges related to balancing participant privacy with data quality when using online recruitment and data collection.

[25] Ridge D, Bullock L, Causer H, Fisher T, Hider S, Kingstone T, Gray L, Riley R, Smyth N, Silverwood V, Spiers J, Southam J (2023). 'Imposter participants' in online qualitative research, a new and increasing threat to data integrity?. Health Expect.

[26] Pellicano E, Adams D, Crane L, Hollingue C, Allen C, Almendinger K, Botha M, Haar T, Kapp SK, Wheeley E (2023). Letter to the editor: a possible threat to data integrity for online qualitative autism research. Autism.

[27] Salinas MR (2023). Are your participants real? Dealing with fraud in recruiting older adults online. West J Nurs Res.

[28] Pozzar R, Hammer MJ, Underhill-Blazey M, Wright AA, Tulsky JA, Hong F, Gundersen DA, Berry DL (2020). Threats of bots and other bad actors to data quality following research participant recruitment through social media: cross-sectional questionnaire. J Med Internet Res.

[29] Chandler J, Sisso I, Shapiro D (2020). Participant carelessness and fraud: consequences for clinical research and potential solutions. J Abnorm Psychol.

[30] Strickland JC, Stoops WW (2020). Utilizing content-knowledge questionnaires to assess study eligibility and detect deceptive responding. Am J Drug Alcohol Abuse.

[31] Rennick-Egglestone S, Elliott R, Smuk M, Robinson C, Bailey S, Smith R, Keppens J, Hussain H, Pollock K, Cuijpers P, Llewellyn-Beardsley J, Ng F, Yeo C, Roe J, Hui A, van der Krieke L, Walcott R, Slade M (2020). Impact of receiving recorded mental health recovery narratives on quality of life in people experiencing psychosis, people experiencing other mental health problems and for informal carers: Narrative Experiences Online (NEON) study protocol for three randomised controlled trials. Trials.

[32] Robinson C, Newby C, Rennick-Egglestone S, Llewellyn-Beardsley J, Ng F, Elliott RA, Slade M (2023). Statistical analysis plans for two randomised controlled trials of the Narrative Experiences Online (NEON) Intervention: impact of receiving recorded mental health recovery narratives on quality of life in people experiencing psychosis (NEON) and people experiencing non-psychosis mental health problems (NEON-O). Trials.

[33] Slade M, Rennick-Egglestone S, Elliott R, Newby C, Robinson C, Gavan SP, Paterson L, Ali Y, Yeo C, Glover T, Pollock K, Callard F, Priebe S, Thornicroft G, Repper J, Keppens J, Smuk M, Franklin D, Walcott R, Harrison J, Smith R, Robotham D, Bradstreet S, Gillard S, Cuijpers P, Farkas M, Zeev DB, Davidson L, Kotera Y, Roe J, Ng F, Llewellyn-Beardsley Joy, NEON study group (2024). Effectiveness and cost-effectiveness of online recorded recovery narratives in improving quality of life for people with non-psychotic mental health problems: a pragmatic randomized controlled trial. World Psychiatry.

[34] Merry SN, Stasiak K, Shepherd M, Frampton C, Fleming T, Lucassen MFG (2012). The effectiveness of SPARX, a computerised self help intervention for adolescents seeking help for depression: randomised controlled non-inferiority trial. BMJ.

[35] Health Research Authority SPARX-UK: pilot and feasibility trial.

[36] Lawlor J, Thomas C, Guhin AT, Kenyon K, Lerner MD, Drahota A (2021). Suspicious and fraudulent online survey participation: introducing the REAL framework. Methodological Innov.

[37] Teitcher JEF, Bockting WO, Bauermeister JA, Hoefer CJ, Miner MH, Klitzman RL (2015). Detecting, preventing, and responding to "fraudsters" in internet research: ethics and tradeoffs. J Law Med Ethics.

[38] Gravel E, Gelly MA, Sansfaçon AP (2024). Dealing with scam in online qualitative research strategies and ethical considerations.

[39] Liu E, Sun L, Bellon A, Voelker G, Savage S, Munyaka I Understanding the viability of Gmail's origin indicator for identifying the sender.

[40] Wilson PM, Petticrew M, Calnan M, Nazareth I (2010). Effects of a financial incentive on health researchers' response to an online survey: a randomized controlled trial. J Med Internet Res.

[41] Bromberg J, Wood ME, Black RA, Surette DA, Zacharoff KL, Chiauzzi EJ (2012). A randomized trial of a web-based intervention to improve migraine self-management and coping. Headache.

[42] Bauermeister JA, Pingel E, Zimmerman M, Couper M, Carballo-Diéguez A, Strecher VJ (2012). Data quality in web-based HIV/AIDS research: handling invalid and suspicious data. Field methods.

