# Correction: Data Integrity Issues With Web-Based Studies: An Institutional Example of a Widespread Challenge

**DOI:** 10.2196/67286

**Published:** 2024-10-17

**Authors:** Blandine French, Camilla Babbage, Katherine Bird, Lauren Marsh, Mirabel Pelton, Shireen Patel, Sarah Cassidy, Stefan Rennick-Egglestone

**Affiliations:** 1 School of Psychology University of Nottingham Nottingham United Kingdom

In “Data Integrity Issues With Web-Based Studies: An Institutional Example of a Widespread Challenge” (JMIR Ment Health 2024;11:e58432) 4 errors have been noted:

1. In *Introduction*, the reference range in the sentence "These issues have also been reported in qualitative web-based interviews [2-26]..." has been revised to:

These issues have also been reported in qualitative web-based interviews [24-26]...

2. In the subsection *Case Study 4: Web-Based Survey to Assess the Acceptability of SPARX, What Happened?*, the age range in the sentence "Our target was to reach 100-200, 1- to 19-year-olds" has been revised to:

Our target was to reach 100-200, 11- to 19-year-olds.

3. In the subsection *Methodological or Research Challenges and Strategies, Overview*, the reference range in the sentence "This includes many strategies that have been summarized in publications [3-38]..." has been revised to:

This includes many strategies that have been summarized in publications [36-38]...

4. Also in the subsection *Methodological or Research Challenges and Strategies, Overview*, the reference range in the sentence "There are many noteworthy strategies put forward in this literature [3-38]…” has been revised to:

There are many noteworthy strategies put forward in this literature [36-38]...

The correction will appear in the online version of the paper on the JMIR Publications website on October 17, 2024, together with the publication of this correction notice. Because this was made after submission to PubMed, PubMed Central, and other full-text repositories, the corrected article has also been resubmitted to those repositories.

