# Supplementary material for: Data Integrity Issues With Web-Based Studies: An Institutional Example of a Widespread Challenge
Source: JMIR Ment Health. 2024 Sep 16;11:e58432. doi: 10.2196/58432 (PMC11443183; doi:10.2196/58432)
Supplement: Multimedia Appendix 1 [file mental_v11i1e58432_app1.docx]

NEON study repeat registration procedures

# Background

The NEON study is currently running three clinical trials of the NEON Intervention, which delivers access to recorded recovery narratives in the NEON Collection. The NEON Trial is a definitive trial looking at whether receiving access to the NEON Intervention benefits people with experience of psychosis. The NEON-O and NEON-C trials are exploratory/feasibility trials generating knowledge to support future definitive trials of the use of the NEON Intervention for people with non-psychosis mental health problems / who are carers for people with mental health problems respectively.

This document outlines NEON study procedures for identifying and responding to suspected repeat registrations from the same trial participant. Repeat registrations are a known feature of trials of online health interventions and can result in loss of power through loss to follow-up (1), but little guidance is available on procedures for managing repeat registration.

Decisions on how to respond to suspected repeat registrations will need to strike a balance between three ethical imperatives:

1. meeting our duty-of-care to avoid clinical harm to a vulnerable population
2. maximising the scientific quality and integrity of the trial to ensure the results are meaningful
3. (for repeat registrations on the NEON Trial where participants are compensated for completion of baseline and follow-up questionnaires) avoiding mis-use of taxpayer’s money if repeat registrations lead to multiple voucher payments

Procedures outlined in this document have been developed to allow for a defensible decision to be made about possible repeat registrations, including if uncertainty is present (for example if a participant fails to respond to a request for information about whether a repeat registration has occurred), and for auditing of the decision-making process to be conducted.

The scope of this document is all three NEON trials. Document versioning will be managed in accordance with PCTU_SOP_QA 03_Document Control_V 6.0. The NEON study Programme Steering Committee (PSC) decided at a meeting on 11.9.20 (minuted in TMF) that this document to be sent to review by all members once complete, with a response required from the PSC chair.

# Document history

| **Version** | **Change** |
| --- | --- |
| 0.1 and 0.2 | Incomplete drafts. |
| 0.3 | First complete version, developed by Stefan Rennick-Egglestone (Trial Manager) and Mike Slade (Chief Investigator) taking account of discussion with Programme Steering Committee (PSC) on 7.7.20, NEON Trial Management Group (TMG) on 30.7.20 |
| 0.4 | Additional detail on procedures around CPMS and end-of-study, with comments from Clare Robinson integrated. |
| 0.5 | Updated detail on CPMS reporting. Assessment section re-ordered for clarity. |
| 0.6 | Added a category of “Repeat registration due to confusion” |
| 0.7 | Feedback integrated from Kamela Aliaj (PCTU QA lead), Ginette Taylor (Nottinghamshire Healthcare NHS Foundation Trust administrator), Hannah Nudds and Lauren Matthews (NEON study administrators), Mark Howells (study sponsor), and Chris Newby (NEON statistician). |
| 1.0 | Final feedback integrated from CI, producing a CI-approved version. |

# Glossary

| **Term** | **Definition** |
| --- | --- |
| Repeat registration | An additional registration by a person who has already registered for one of the NEON trials. This would include a registration for an additional trial by someone who has already registered for a first, as inclusion criteria have been structured to only allow participation in a single trial. |
| Repeat registration for access | A repeat registration in order to access the intervention |
| Repeat registration for profit | A repeat registration in order to obtain additional participant payments |
| Repeat registration due to confusion | A repeat registration caused by a participant being confused by trial procedures, and believing that an additional registration was the correct action |
| Related registration | An existing registration which is matched to the registration under consideration, e.g. similar email address, similar participant name, identical IP address |
| Risk indicator | Indicators that a registration might be a repeat registration |
| Similar email address | Two email addresses which are similar, such as:  bob.fish@gmail.com and bob.fish@outlook.com  cherry.blossom@gmail.com and cherry.blossom2@outlook.com |
| Similar participant name | Two names which are similar. Names may be contained in an email sent to the study and/or provided by a participant’s email system, e.g. dunc.donut@gmail.com and Duncan D’onut <orange@big.net> |
| Repeat Registration report | Document collecting information required to allow decision-making about a repeat registration |

# Monitoring for suspected repeat registrations

Administrators employed by Nottinghamshire Healthcare NHS Foundation Trust (NottsHCT) and the University of Nottingham (UoN) will monitor for suspected repeat registrations. Administrators employed by UoN are part of the NEON study team, and hence referred to as “NEON administrators” throughout. Only those accounts with a validated email address should be monitored, as the NEON protocol indicates that consent procedures are not complete until an email address has been validated.

NottsHCT administrators are responsible for making voucher payments only. They will identify possible repeat registrations based on access to information relevant to voucher payments. This consists of: participant email, date and time of voucher request, and IP address of device used to make voucher request. NottsHCT administrators can compare information about voucher requests between participants so as to identify unusual patterns. NottsHCT administrators should not examine public records relating to the participant. NottsHCT administrators will monitor for repeat registrations each time a batch of voucher requests is processed. For the NEON Trial, approximately 2500 voucher requests will be received, and hence monitoring for repeat registrations may require the use of automated tools such as spreadsheets.

NEON administrators conduct all other administrative actions on the study. They will identify possible repeat registrations based on access to relevant profile information collected about the participant. Collected information will be reviewed at least weekly. Some relevant profile information will be collected automatically by the NEON web application at the following participant interaction events: email address validation, completion of trial survey set (baseline, week 1, week 12, week 52), request of voucher, provision of safety report. At each milestone, the following will be considered relevant: email address, participant ID, date and time of milestone, IP address of device used to reach milestone. If the participant has been randomised, treatment allocation is considered relevant, as in a repeat registration for access, the final registration will be for an account allocated to the intervention group. If the participant has completed the quality of life questionnaire at a timepoint, then the total MANSA score and time taken to complete the MANSA questionnaire is considered relevant, as very low or high scores or excessively rapid responses might indicate a response set has been provided.

If someone sends the study team or NottsHCT administrators an email from an email address used for a registration, then this is considered part of the profile, including all email header information.

# Generation of Repeat Registration Reports

NEON administrators will maintain Repeat Registration reports that summarise all relevant information about included accounts, and which are used to enable assessment and decision making. Since Repeat Registration reports can include treatment allocation and hence are unblinding, then they should be stored and maintained on the NEON Trial File Store.

An account is added into a Repeat Registration report if one or more risk indicators are met (see below). Initial partial versions of Repeat Registration reports can be generated by NottsHCT administrators including details of voucher requests only, but these reports then need to be completed by NEON administrators. Where the contents of Repeat Registration reports impact on voucher payments, then they should be shared with NottsHCT administrators. Since NottsHCT administrators do not have access to the NEON Trial File Store, then any sharing of repeat registration reports should be by email attachment, with all document strictly password protected.

If emails are included in the profile of information presented in a Repeat Registration Report, then the email should be stored in the same folder by dragging and dropping the email itself on the NEON Trial File Store. This preserves email headers which might provide information useful to the assessment process. If emails are received by NottsHCT administrators, they should be forwarded to NEON administrators for storage as above. If a text or voicemail is received from a participant where the phone number links to an email address included in a Repeat Registration Report, then the text and date/time of receiving the message should be stored alongside the Repeat Registration Report. Any communication received should be summarised in the Repeat Registration Report (e.g. by including the filename containing an email received from a participant).

Since effective decision making requires knowledge about all accounts that might be controlled by a participant, then if a related account is already included in an existing report, the account is added to the report that contains the related account. Otherwise, a new report is generated, given a unique ID number, and placed into a numbered sub-folder in the Repeat Registration folder on the NEON Trial file store. If subsequent information is received that links multiple repeat registration reports, then the relevant reports will be merged into a single report, retaining only the report with the lowest number. For example, if the IP address for a week 12 voucher request for a participant in repeat registration report 7 matches the IP address for a week 1 voucher request for a participant in repeat registration report 9, then all information in repeat registration report 9 would be merged into report 7, and report 9 should be deleted . Numbers should not be reused.

The current list of risk indicators (which may be extended) comprises:

1. Email address similar to existing registration
2. Participant name similar to existing registration
3. Match on IP address with a suspected or confirmed repeat registration, or to any account related to the suspected or confirmed repeat registrations
4. Match on IP address with three or more other registrations
5. Any other bespoke reason documented in the repeat registration report by NottsHCT or NEON administrators for the purpose of audit

If there is uncertainty in a decision about whether names or email address are similar, then the account should be added to a Repeat Registration report. Only a precise match on IP address should be considered for risk indicators 3 and 4, i.e. these risk indicators are not present if IP addresses are different but similar. If a registration is added to a Repeat Registration Report, then any pending voucher payments should be suspended pending assessment.

# Assessment of Repeat Registration Reports

Once an account has been added to a Repeat Registration Report, it should be assessed by the Chief Investigator or delegate (the **assessor**), who will decide the next steps to be taken. All decisions about the next steps to take made should be entered into the Repeat Registration report and date-stamped, with sufficient detail to allow next steps to be conducted. Each account in a Repeat Registration report can be assessed multiple times, with further assessments being triggered if additional information becomes available that might challenge a decision (such as a new IP address being used to complete week 12 survey results).

If the assessor is not the CI, and if a Repeat Registration report needs to be discussed with the CI, e.g. if there are clinical concerns about a participant, then wherever possible blinding will be maintained through the preparation of a redacted version. However, if an effective decision depends upon information that would unblind the CI, and if a particular case indicates significant clinical or trial integrity concerns, then the information will not be redacted, and the rationale for not redacting the information will be recorded in the TMF for purposes of audit along with the number of the repeat registration report. This is in keeping with procedures for “emergency unblinding” described in the NEON protocol.

Ideally, accounts should be examined and decisions about next steps made within 2 working days of being added to a report, so that vouchers can still be paid within the five working day window specified in the Participant Information Sheet if the account is deemed to be “not at risk”. However, trial integrity and clinical considerations are considered more important than timely voucher payments, and decisions about next steps will be taken more slowly if additional time is needed.

In addition to information held in the Repeat Registration reports, the sponsor has confirmed (email 22.9.20) that the assessor can consult public sources of information for evidence of clinical vulnerability, such as by searching for public statements made by the participant on Twitter. Any relevant information should be added to the Repeat Registration report, with the source specified for future reference. Public information collected about clinical vulnerability will only be used to avoid harm to the participant, e.g. to inform a choice about whether emails should be sent to participants or not, or informing decisions about whether one account should be allowed to continue in the case of a registration for access.

At initial assessment and all subsequent assessment, the assessor should allocate one of the following statuses to the account being examined:

- Account not at risk
- Account at risk
- Suspension confirmed – repeat registration for access
- Suspension confirmed – repeat registration due to confusion
- Suspension confirmed – repeat registration for profit
- Suspension confirmed – [specify other reason]
- Account continues – repeat registration for access
- Account continues – repeat registration due to confusion
- Account continues – [specify other reason]

Even if allocated the status “account not at risk”, the account is not removed from the Repeat Registration Report, as additional information may become available to change this status. Until a decision has been made to suspend an account, all normal study contact apart from voucher payment should continue (such as reminders to complete study questionnaires or intervention engagement emails).

If an account is “at risk”, then further investigatory actions should be planned and documented in the Repeat Registration report. This might include information seeking actions such as contacting the participant, and specific dates by which decisions are needed. In most cases, a participant will be sent an information seeking email before a decision is made, asking for a response within one week. The NEON Lived Experience Advisory Panel (LEAP) have advised at a meeting on 1.10.20 that all communication sent to participants should avoid sounding accusatory, annoyed or threatening. All communication should be written on the assumption that the recipient is vulnerable.

The Repeat Registration report should include a timestamped record of all emails sent, and emails should be copied into the same folder as the Repeat Registration Report. Standard emails are preferred in most cases since the development of standard emails allows for the refinement of effective text. They should be named, versioned and dated, and stored in the Trial Management Documents section of the TMF. Alternatively, a bespoke email can be written.

In some cases an email will not be sent. Anticipated circumstances include clinical concerns being present to the extent that an email might cause harm, or collected information indicating so strongly that a repeat registration has occurred that participant responses to information seeking emails would be unlikely to influence a decision-making process, such as if a repeat registration appeared to be by someone whose accounts had previously been suspended.

Voucher payments are only allowed for those account identified as “Not at risk” or any variant of “Account continues”. For clarity of communication with NottsHCT administrators, the Repeat Registration Report will explicitly state whether voucher payment is currently allowed, suspended, or not relevant. “Not relevant” is reserved for the NEON-O and NEON-C Trials where vouchers are not being paid. Where a series of decisions have been made that alter voucher payment status, the most recent decision (and hence the active one) will be colour coded to reduce risk of error.

# Decisions to suspend accounts

Once the assessment has been completed, the assessor will make a judgement based on the balance of probabilities and using all available evidence. The default assumption will be that the account is not a repeat registration, so positive evidence will be needed to suspend an account. Where there is relative certainty about the existence of a repeat registration but significant uncertainty about whether the registration was for access, due to confusion, or for profit, the decision will be that it is registration for access or due to confusion. A description of the case, the decision made and the rationale for the decision made should be added to a “difficult decisions log”, to support consistency of decision-making. Example case description: “Seven accounts sharing an IP address, with no response from any account to a standard initial email. Suspension confirmed: repeat registration for profit”.

Whenever a decision is made to suspend an account, the assessor should document their reasons for making a specific decision in the Repeat Registration Report, and should change its status to one of the variants of “Suspension confirmed”. Differentiating between different types of repeat registration is important because different analysis principles might be applied to accounts suspended for different reasons. Decisions on analysis principles will be documented in the Statistical Analysis Plan (SAP).

Since by definition more than one account must be involved in an instance of repeat registration, then each decision to suspend an account must also involve a decision about at least one related account.

If an account is suspended due to a repeat registration for access, and if a participant is considered clinically vulnerable, then the first account obtaining access to the intervention will be allowed to continue, and all other related accounts will be suspended. The continuing account would be give the status “Account continues – repeat registration for access”.

If an account is suspended due to a registration caused by confusion, then any account giving access to the intervention will be allowed to continue, and if all accounts have been allocated to the control group then the first account will be selected to continue.

As a standard procedure, all accounts where it has been decided that an attempt to register for profit will be given the status “Suspension confirmed – registration for profit”. This is to discourage further repeat registrations for profit.

## Informing participants of suspension

In the event that an account is suspended, an email will normally be sent to inform the participant of this, unless clinical or other considerations are present which means this email might cause harm. A standard email can be used, which might have a different form in the case of Registration for Profit (where the email might be used to discourage further registration) and Registration for Access (where a participant needs to understand that one account is being permitted to continue.

Following guidance from the NEON study Programme Steering Committee (PSC) (meeting on 7.7.20, discussion minuted in TMF) an email used to notify suspension on the grounds of “registration for profit” should not indicate that the reason is registration for profit, as this would amount to an accusation of fraud, and the study team does not have the legal expertise or investigative powers to make such an accusation valid. Instead, notification emails might indicate suspension based on a need to preserve trial integrity, but will explicitly indicate that the participant should not register again.

In any email informing the participant of suspension, no appeals process will be mentioned. There will be no appeals process because the default assumption is that an account is not a repeated registration, thus reducing the chance of erroneous suspension, and because of the risks to trial integrity of trial staff responding to vexatious appeals.

## Processing a suspension

Once an email has been sent, then a NEON administrator will use the NEON web-application management interface to suspend the account. This should block the user from logging in and stop the sending of automated emails, such as those notifying a participant that follow-up questionnaires are due. Following a discussion in the NEON study Trial Management Group (TMG) (meeting on 24.8.20, discussion minuted in TMF) it was decided that suspensions are final, as from the point of suspension, the trial data set for the suspended participant will begin to have missing data. In the event that a suspended participant queries a suspension, the study team may choose to have documented discussions with them for the purpose of updating repeat registration procedures.

## Relationship to informed consent procedures

In the context of informed consent procedures, a suspended participant remains a participant, so retains the right to confidentiality and withdrawal set out in the Participant Information Sheet. Once suspended, a participant cannot withdraw themselves using automated functionality provided by the NEON web application, since they will have lost access to the NEON web application. Instead, they would need to submit a withdrawal request to the NEON team by emailing [neon@nottingham.ac.uk](mailto:neon@nottingham.ac.uk).

## Reporting and monitoring

The NEON protocol (v5 17.5.20) indicates that “reasons for suspension will be reported to the study sponsor”. This will be interpreted as follows: 1. a quarterly report will be provided to the sponsor summarising numbers of suspended accounts per trial 2. repeat registration reports will document reasons for suspension 3. repeat registration reports contain information that would unblind the sponsor, and hence will not be available until the NEON senior statistician indicates that unblinding information can be shared 4. If requested by the study sponsor, the trial monitor can assess repeat registration reports, and send summaries to the study sponsor.

# Reporting of participation to CPMS

Study participation is reported to the Central Portfolio Management System (CPMS), normally on a monthly basis through upload of a spreadsheet. Reporting is through upload of a spreadsheet by the study co-ordinator or delegate. CPMS has quarterly checkpoints (31.3, 30.6, 30.9, 31.12), for which all recruitment in the three months up to the checkpoint should be finalised. Checkpoints at the end of June, September and December are used to drive reporting to funder for the purposes of study monitoring. The checkpoint end of March is additionally used to drive annual payments to research sites for service support costs, and to the lead R&D site for Excess Treatment Costs.

The following principles and processes were agreed with the LCRN through a telephone conversation on 21.9.20:

- accounts suspended due to repeat registration do not count as study participation and hence should not be included in the CPMS database by study end. If an account has been added to CPMS and is subsequently suspended, a request should be sent to [crncc.portfoliob@nihr.ac.uk](mailto:crncc.portfoliob@nihr.ac.uk) to ask for its removal. This can be done at any point up until study end, but is significantly easier to process if done before end of quarter ending 31.3
- to reduce the risk of reporting to CPMS accounts that are subsequently suspended, then for monthly reports, if a participant has not previously been reported to CPMS, and is included in a repeat registration report, then it should only be reported to CPMS if the participant has been allocated the status of “Account continues – registration for access”. This means that even accounts given the status of “Not at risk” should not be reported, since some accounts initially allocated the status “not at risk” become “at risk” when further information becomes available.
- To enable external monitoring of study recruitment, then for each quarterly checkpoint, then as much as possible, decisions should be made about the final status of accounts included in repeat registration reports should be made, but if uncertainty remains, an account should not be reported.
- Before end of the trial recruitment period, decisions should be made on all accounts included in repeat registration reports.

# Processes near to the end of the recruitment period for the trials

Suspended accounts do not count as attrition, as otherwise large scale attempts to create repeat accounts would lead to insufficient analysable participants. Near the predicted end of the trial, the point at which recruitment should be closed will be reassessed on a weekly basis, to take into account remaining uncertainty as to which accounts will form part of the final trial dataset. The implication of this is that, for each trial, spare numbers should be included in the randomisation list. The study co-ordinator will monitor the length of the current randomisation list to ensure that it is of sufficient length.

# Implications for trial resources

Cases of repeat registrations can reduce trial resources, since they are not a form of attrition and since vouchers might have been paid to accounts that are subsequently suspended, such as if information collected at week 12 follow-up indicates a decision that a repeat registration has occurred. On a quarterly basis, the study co-ordinator will assess lost resources, and identify any risks to the NEON Trial.

# References

1. Mathieu E, McGeechan K, Barratt A, Herbert R. Internet-based randomized controlled trials: a systematic review. Journal of the American Medical Informatics Association. 2013;20(3):568-76.
