# Supplementary material for: Data Integrity Issues With Web-Based Studies: An Institutional Example of a Widespread Challenge
Source: JMIR Ment Health. 2024 Sep 16;11:e58432. doi: 10.2196/58432 (PMC11443183; doi:10.2196/58432)
Supplement: Multimedia Appendix 2 [file mental_v11i1e58432_app2.docx]

|  | **BASELINE voucher request** | **Week 1 voucher request** | **Week 12 voucher request** | **Status** |
| --- | --- | --- | --- | --- |
| <Registered email>  <Trial name>: <Treatment>  User ID: <ID> | Received <date>, <time>  Paid <date>  *IP: <request IP>* | Received <date>, <time>  *Voucher not paid in line with decision <date 3>* | Not yet relevant | **Decision <date 1>**  Not at risk. Vouchers to be paid  **Decision <date 2>**  Account at risk.  Initial email v2 31.7.20 sent <date> [initials of sender]  Vouchers suspended pending response.  **Decision <date 3>**  Suspension confirmed – registration for profit  Reason: <describe>  No further vouchers. |

# Notes

Colour coding can be used to highlight similarities in email or matching IP addresses.

All historical decisions retained as audit log.

Colour coding used to highlight current decision on voucher receipt.

Status column can be used to highlight other relevant information, such as any related Serious Adverse Events..
